# Berry-Esseen bounds of weighted kernel estimator for a nonparametric regression model based on linear process errors under a LNQD sequence

**DOI:** 10.1186/s13660-017-1604-8

**Published:** 2018-01-08

**Authors:** Liwang Ding, Ping Chen, Yongming Li

**Affiliations:** 1 School of Science, Nanjing University of Science and Technology, Nanjing, 210094 P.R. China; 2 School of Information and Statistics, Guangxi University of Finance and Economics, Nanning, 530003 P.R. China; 3 School of Mathematics and Computer Science, Shangrao Normal College, Shangrao, 334001 P.R. China

**Keywords:** 60G05, 60G20, O212.7, weighted kernel estimator, LNQD sequence, Berry-Esseen bound, linear process

## Abstract

In this paper, the authors investigate the Berry-Esseen bounds of weighted kernel estimator for a nonparametric regression model based on linear process errors under a LNQD random variable sequence. The rate of the normal approximation is shown as $O(n^{-1/6})$ under some appropriate conditions. The results obtained in the article generalize or improve the corresponding ones for mixing dependent sequences in some sense.

## Introduction

We discuss that the estimation of the fixed design nonparametric regression model involves a regression function $g(\cdot )$ which is defined on a closed interval $[0,1]$:

1.1 $$ Y_{i}=g(t_{i})+\varepsilon_{i}\quad (1\leq i\leq n), $$

where $\{t_{i}\}$ are known fixed design points, we suppose $\{t_{i}\}$ to be ordered $0\leq t_{1}\leq \cdots \leq t_{n}\leq 1$, and $\{\varepsilon_{i}\}$ are random errors.

As we all know, model (1.1) has been considered extensively by many authors, e.g., Schuster and Yakowitz [1] studied the nonparametric model (1.1) with i.i.d. errors. They obtained the strong convergence and asymptotic normality of the estimator of $g(\cdot )$, and Qin [2] obtained the strong consistency of the estimator of $g(\cdot )$. Yang [3–5] studied the nonparametric model (1.1) with *φ*-mixing errors, censored data random errors and negatively associated errors. He obtained the complete convergence, strong consistency and uniformly asymptotic normality of the estimator of $g(\cdot )$, respectively. Zhou *et al.* [6] studied the nonparametric model (1.1) with weakly dependent processes. They obtained the moment consistency, strong consistency, strong convergence rate and asymptotic normality of the estimator of $g(\cdot )$, etc. Inspired by the literature above, we are devoted to investigating the Berry-Esseen bounds of the estimator for linear process errors in the nonparametric regression model (1.1).

In the article, we will discuss the Berry-Esseen bounds of the estimator of $g(\cdot )$ in the model (1.1) with repeated measurements. Here, we recall the weighted kernel estimator of nonparametric regression functions. A popular nonparametric estimate of $g(\cdot )$ is then

1.2 $$ g_{n}(t)=\sum_{i=1} ^{n}Y_{i} \frac{t_{i}-t_{i-1}}{h_{n}}K \biggl( \frac{t-t_{i}}{h_{n}} \biggr) , $$

where $K(u)$ is a Borel measurable function, $0< h_{n}\to 0$ as $n\to \infty $.

The weighted kernel estimator was first proposed by Priestley and Chao [7], who discussed the weak consistency conditions of $g(\cdot )$, and subsequently it has been studied extensively by many authors. For instance, in the independent assumption, Benedetti [8] gave the sufficient condition for the strong consistency of $g(\cdot )$ under the condition of $\mathrm{E}\varepsilon_{1}^{4}<\infty $. Schuster and Yakowitz [1] discussed the uniformly strong consistency of $g(\cdot )$. Qin [2] extended the moment condition to $\mathrm{E} \vert \varepsilon_{1} \vert ^{2+\delta }<\infty $ (as ${\delta >0}$). Under the mixing dependent assumption, Yang [3] and [9] not only comprehensively improved these results under *φ*-mixing and *ρ*-mixing, but reduced the condition to $\sup_{i} {\mathrm{E}} \vert \varepsilon_{i} \vert ^{r}<\infty $ (as $r>1$), weakened the addition of the kernel function $K(\cdot )$. Pan and Sun [10] extended this discussion to censored data and gave some sufficient conditions for strong consistency in the independent and *φ*-mixing case. Yang [4] discussed the consistency of weighted kernel estimators of a nonparametric regression function with censored data and obtained strong consistency under some more weakly sufficient conditions. But, up to now, there have been few results related to weighted kernel estimator for model (1.1) with linear process errors.

The Berry-Esseen theorem is the rate of convergence in the central limit theorem. There is a lot of literature regarding this kind of the Berry-Esseen bounds theorem. For the details, Cheng [11] established a Berry-Esseen type theorem showing the near-optimal quality of the normal distribution approximation to the distribution of smooth quantile density estimators. Wang and Zhang [12] obtained a Berry-Esseen type estimate for NA random variables with only finite second moment. They also improved the convergence rate result in the central limit theorem and precise asymptotics in the law of the iterated logarithm for NA and linearly negative quadrant dependent sequences. Liang and Li [13] derived the Berry-Esseen type bound based on linear process errors under negatively associated random variables. Li *et al.* [14] established the Berry-Esseen bounds of the wavelet estimator for a nonparametric regression model with linear process errors generated by *φ*-mixing sequences. Yang *et al.* [15] investigated the Berry-Esseen bound of sample quantiles for NA random variables, the rate of normal approximation is shown as $O(n^{-1/9})$, etc.

In this paper, we shall study the above nonparametric regression problem with linear process errors generated by a linearly negative quadrant dependent sequence.

### Definition 1.1

([16])

Two random variables *X* and *Y* are said to be negative quadrant dependent (NQD in short) if, for any $x, y\in \mathbf{R}$,

$$ {\mathrm{P}}(X< x, Y< y)\leq {\mathrm{P}}(X< x)\mathrm{P}(Y< y). $$

### Definition 1.2

([17])

A sequence $\{X_{n}, n\geq 1\}$ of random variables is said to be linearly negative quadrant dependent (LNQD in short) if for any disjoint subsets $\mathbf{A}, \mathbf{B} \in \mathbf{Z}^{+}$ and positive $r'_{i}s$, $\sum_{i\in \mathbf{A}}r _{i}X_{i}$ and $\sum_{j\in \mathbf{B}}r_{j}X_{j}$ are NQD.

The concept of LNQD sequence was introduced by Newman [17], who investigated the central limit theorem for a strictly stationary LNQD process, and it subsequently has been studied by many authors. Wang and Zhang [12] provided the uniform rates of convergence in the central limit theorem for LNQD random variables. Ko *et al.* [18] established the Hoeffding-type inequality for a LNQD sequence. Ko *et al.* [19] discussed the strong convergence and central limit theorem for weighted sums of LNQD random variables. Wang *et al.* [20] presented some exponential inequalities and complete convergence for a LNQD sequence. Wang and Wu [21] gave some strong laws of large numbers and strong convergence properties for arrays of rowwise NA and LNQD random variables. Li *et al.* [22] established some inequalities and asymptotic normality of the weight function estimate of a regression function for a LNQD sequence. Shen *et al.* [23] investigated the complete convergence for weighted sums of LNQD random variables based on the exponential bounds and obtained some complete convergence for arrays of rowwise LNQD random variables, etc.

However, there are very few literature works on Berry-Esseen bounds of weighted kernel estimator for nonparametric regression model (1.1) with linear process errors. So, the main purpose of the paper is to investigate the Berry-Esseen bounds of weighted kernel estimator for nonparametric regression model with linear process errors generated by a LNQD sequence.

In what follows, let *C* be positive constants which may be different in various places. All limits are taken as the sample size *n* tends to ∞, unless specified otherwise.

The structure of the rest of the paper is as follows. In Section 2, we give some basic assumptions and main results. Some preliminary lemmas are stated in Section 3. Proofs of the main results are provided in Section 4. Authors’ declaration is given at the end of the paper.

## Assumptions and main results

In order to facilitate the process, we write $\rho_{n}^{2}:=\rho_{n} ^{2}(t)=\operatorname {Var}(\widehat{g}_{n}(t))$, $U_{n}:=U_{n}(t)=\sigma_{n} ^{-1} \{\widehat{g}_{n}(t)-\mathrm{E}\widehat{g}_{n}(t)\}$, $V(n)=O(n ^{-(r-2)(r+\delta )/2\delta })$, $V(q)=\sup_{j\geq 1}\sum_{j: \vert j-i \vert \geq q} \vert \operatorname {Cov}(\varepsilon_{i}, \varepsilon_{j}) \vert $, $\delta_{n}= \max_{1\leq i\leq n}(t_{i}-t_{i-1})$.

First, we make the following basic assumptions:

1. $\{\varepsilon_{j}\}_{j\in \mathbf{Z}}$ has a linear representation $\varepsilon_{j}=\sum_{k=-\infty }^{\infty }a_{k}e_{k-j}$, where $\{a_{k}\}$ is a sequence of real numbers with $\sum_{k=-\infty }^{ \infty } \vert a_{k} \vert <\infty $, $\{e_{j}\}$ is a strictly stationary and LNQD sequence with $\mathrm{E}e_{j}=0$, $\mathrm{E} \vert e_{j} \vert ^{r}<\infty $ for some $r>2$, and $V(1)=\sup_{j\geq 1}\sum_{j: \vert j-i \vert \geq 1} \vert \operatorname {Cov}(\varepsilon_{i}, \varepsilon_{j}) \vert <\infty $;
2. $g(\cdot )$ satisfies the Lipschitz condition of order *α* ($\alpha >0$) on $[0, 1]$, $K(\cdot )$ satisfies the Lipschitz condition of order *β* ($\beta >0$) on $\mathbf{R}^{1}$, $\int^{+\infty }_{-\infty }K(u)\,du=1$, $\int^{+\infty }_{-\infty } \vert K(u) \vert \,du< \infty $, where $K(\cdot )$ is bounded on $\mathbf{R}^{1}$;
3. $h_{n}\to 0$ and $\delta_{n}\to 0$ as $n\to \infty $, $\frac{1}{h_{n}} ( ( \frac{\delta_{n}}{h_{n}} ) ^{\beta }+ \delta_{n}^{\alpha } ) \to 0$ as $n\to \infty $, let $\frac{\delta _{n}}{h_{n}}=O(n^{-\theta })$ for $\theta >0$;
4. $\max_{1\leq i\leq n}\frac{x_{i}-x_{i-1}}{h_{n}}K ( \frac{x-x_{i}}{h_{n}} ) =O(\rho_{n}^{2}(t))\leq O ( \frac{ \delta_{n}}{h_{n}} ) =O(n^{-\theta })$;
5. There exist two positive integers *p* and *q* such that $p+q\leq 3n$, $qp^{-1}\to 0$, and $\xi_{in}\to 0$ ($i=1,2,3,4$), where $\xi_{1n}=n^{1-\theta }qp^{-1}$, $\xi_{2n}=pn^{-\theta }$, $\xi_{3n}=n ( \sum_{ \vert j \vert >n} \vert a_{j} \vert ) ^{2}$, $\xi_{4n}=p^{r/2-1}n^{(1-r/2) \theta }$.

### Remark 2.1

(A1) is a basic condition of the LNQD sequence, and conditions (A2)-(A3) are general assumption conditions of the weighted kernel estimator which have been used by some authors such as Yang [3, 4, 9], Pan and Sun [10].

### Remark 2.2

Let $K(\cdot )$ be bounded, suppose that conditions (A2)-(A3) hold true. We have

$$ \max_{1\leq i\leq n}\frac{x_{i}-x_{i-1}}{h_{n}}K \biggl( \frac{x-x _{i}}{h_{n}} \biggr) \leq \max_{1\leq i\leq n}\frac{x_{i}-x_{i-1}}{h _{n}} \biggl\vert K \biggl( \frac{x-x_{i}}{h_{n}} \biggr) \biggr\vert \leq O \biggl( \frac{ \delta_{n}}{h_{n}} \biggr) =O\bigl(n^{-\theta }\bigr). $$

Thus, (A4) can be assumed.

### Remark 2.3

For (A5), $\xi_{in}\to 0$, $i=1,2,3,4$, are easily satisfied, if *p*, *q* are chosen reasonable, which is the same as in Yang [5] and Li *et al.* [14]. So, (A5) is a standard regularity condition used commonly in the literature.

Therefore, we can see that conditions (A1)-(A5) in this paper are suitable and reasonable.

Next, we give the main results as follows.

### Theorem 2.1

*Assume that* (A1)-(A5) *hold true*, *then for each* $t\in [0,1]$, *we can get that*

$$ \sup_{y} \bigl\vert {\mathrm{P}}\bigl(U_{n}(t) \leq y\bigr) -\Phi (y) \bigr\vert \leq C \bigl\{ \xi _{1n}^{1/3}+ \xi_{2n}^{1/3}+ \xi_{3n}^{1/3}+ \xi_{4n} + V^{1/3}(q) \bigr\} . $$

### Corollary 2.1

*Assume that* (A1)-(A5) *hold true*, *then for each* $t\in [0,1]$, *we can get that*

$$ \sup_{y} \bigl\vert {\mathrm{P}}\bigl(U_{n}(t) \leq y\bigr) -\Phi (y) \bigr\vert =\circ (1). $$

### Corollary 2.2

*Assume that* (A1)-(A5) *hold true*, $\frac{\delta_{n}}{h_{n}}=O(n^{-\theta })$ *for some* $\theta >0$, *and* $\sup_{n\geq 1} ( n^{\frac{2\theta \delta +6\theta +3\delta +3}{12+8 \delta }} ) \sum_{ \vert j \vert >n} \vert a_{j} \vert <\infty $ *for some* $\delta > 0$. *We can get that*

$$ \sup_{y} \bigl\vert {\mathrm{P}}\bigl(U_{n}(t) \leq y\bigr) -\Phi (y) \bigr\vert =O \bigl( n^{-\frac{2 \theta \delta +6\theta -\delta -3}{18+12\delta }} \bigr) . $$

*Observe*, *taking* $r=3$ *and* $\theta \approx 1$ *as* $\delta \to 0$, *it follows that* $\sup_{y} \vert {\mathrm{P}}(U_{n}(t)\leq y) -\Phi (y) \vert =O(n ^{-1/6})$.

### Remark 2.4

We develop the weighted kernel estimator methods in the nonparametric regression model (1.1) which are different from estimation methods of Liang and Li [13], Li *et al.* [14]. Our theorem and corollaries improve Theorem 3.1 of Li *et al.* [22] for the case of linear process errors generated by LNQD sequences and also generalize the results of Li *et al.* [14] from linear process errors generated by LNQD sequences to the ones generated by *φ*-mixing sequences. So, our results obtained in the paper generalize and improve some corresponding ones for *φ*-mixing random variables to the case of LNQD setting.

## Some preliminary lemmas

First, we have by (1.1) and (1.2) that

$$\begin{aligned} \begin{aligned} U_{n} &= \rho_{n}^{-1}\sum _{i=1}^{n} \varepsilon_{i} \frac{t _{i}-t_{i-1}}{h_{n}}K \biggl( \frac{t-t_{i}}{h_{n}} \biggr) \\ & =\rho_{n}^{-1}\sum_{i=1}^{n} \frac{t_{i}-t_{i-1}}{h_{n}}K \biggl( \frac{t-t_{i}}{h_{n}} \biggr) \Biggl( \sum _{j=-n}^{n}a_{j}e _{i-j}+\sum _{ \vert j \vert >n}a_{j}e_{i-j} \Biggr) := U_{1n}+U_{2n}, \end{aligned} \end{aligned}$$

where

$$ U_{1n}=\sum_{l=1-n}^{2n} \rho_{n}^{-1} \Biggl( \sum_{i=\max \{1,l-n\}} ^{\min \{n,l+n\}}a_{i-l}\frac{t_{i}-t_{i-1}}{h_{n}}K \biggl( \frac{t-t _{i}}{h_{n}} \biggr) \Biggr) e_{l} :=\sum_{l=1-n}^{2n}X_{nl}. $$

Let $U_{1n}=U_{1n}^{\prime }+U_{1n}^{\prime \prime }+U_{1n}^{\prime \prime \prime }$, where $U_{1n}^{\prime }=\sum_{v=1}^{k}z_{nv}$, $U _{1n}^{\prime \prime }=\sum_{v=1}^{k}z_{nv}^{\prime }$, $U_{1n}^{ \prime \prime \prime }=z^{\prime }_{n k+1}$,

$$ z_{nv}=\sum_{i=k_{v}}^{k_{v}+p-1}X_{ni},\quad\quad z_{nv}^{\prime }= \sum_{i=l_{v}}^{l_{v}+q-1}X_{ni},\quad\quad z_{nk+1}^{\prime }= \sum_{i=k(p+q)-n+1}^{2n}X_{ni}, $$

where

$$\begin{aligned}& k=[3n/p+q], \quad\quad k_{v}=(v-1) (p+q)-n+1, \quad\quad l_{v}=(v-1) (p+q)+p-n+1, \\& v=1,\ldots,k, \end{aligned}$$

then

$$ U_{n}=U_{1n}^{\prime }+U_{1n}^{\prime \prime }+U_{1n}^{\prime \prime \prime }+U_{2n}. $$

Next, we give the following main lemmas.

### Lemma 3.1

([3])

*Let* $K(\cdot )$ *satisfy the Lipschitz condition of order* *β* ($\beta >0$) *on* $\mathbf{R}^{1}$, *and* $\int^{+\infty }_{-\infty }K(u)\,du=1$, $\int^{+\infty }_{-\infty } \vert K(u) \vert \,du< \infty $, *where* $K(\cdot )$ *is bounded on* $\mathbf{R}^{1}$. *Assume that* $h_{n}\to 0$ *and* $\delta_{n}\to 0$ *as* $n\to \infty $, *and* $\frac{1}{h _{n}} ( ( \frac{\delta_{n}}{h_{n}} ) ^{\beta }+\delta_{n} ^{\alpha_{0}} ) \to 0$ *as* $n\to \infty $, *then for any* $\alpha_{0}>0$,

$$ \lim_{n\to \infty }\sum_{i=1}^{n} \frac{x_{i}-x_{i-1}}{h _{n}} \biggl\vert K \biggl( \frac{x-x_{i}}{h_{n}} \biggr) \biggr\vert = \int^{+\infty } _{-\infty } \bigl\vert K(u) \bigr\vert \,du,\quad \forall x\in [0, 1]. $$

### Lemma 3.2

([22])

*Let* $\{X_{j}, j\geq 1\}$ *be a LNQD random variable sequence with zero mean and finite second moment* $\sup_{j \geq 1}{\mathrm{E}}(X^{2}_{j})<\infty $. *Assume that* $\{a_{j}, j\geq 1\}$ *is a real constant sequence satisfying* $a:=\sup_{j\geq 1} \vert a_{j} \vert <\infty $. *Then*, *for any* $r>1$,

$$ \mathrm{E} \Biggl\vert \sum^{n} _{j=1}a_{j}X_{j} \Biggr\vert ^{r}\leq Da^{r}n ^{r/2}. $$

### Lemma 3.3

([22])

*If* $X_{1},\ldots, X_{m}$ *are LNQD random variables with finite second moments*, *let* $\varphi_{j}(t_{j})$ *and* $\varphi (t_{1},\ldots,t_{m})$ *be characteristic functions of* $X_{j}$ *and* $(X_{1},\ldots, X_{m})$, *respectively*, *then for all nonnegative* (*or nonpositive*) *real numbers* $t_{1},\ldots, t_{m}$,

$$ \Biggl\vert \varphi (t_{1},\ldots,t_{m})-\prod _{j=1}^{m}\varphi_{j}(t_{j}) \Biggr\vert \leq 4\sum_{1\leq k\leq l\leq m} \vert t_{k}t_{l} \vert \bigl\vert \operatorname {cov}(X_{k},X_{l}) \bigr\vert . $$

### Lemma 3.4

([14])

*Suppose that* $\{\zeta_{n}: n\geq 1\}$, $\{\eta_{n}: n\geq 1\}$ *and* $\{\gamma_{n}: n\geq 1\}$ *are three random variable sequences*, $\{\xi_{n}: n\geq 1\}$ *is a positive constant sequence*, *and* $\xi_{n}\to 0$. *If* $\sup_{y} \vert F_{\zeta_{n}}(y)-\Phi (y) \vert \leq C\xi_{n}$, *then for any* $\varepsilon_{1}>0$ *and* $\varepsilon_{2}>0$,

$$ \sup_{y} \bigl\vert F_{\zeta_{n}+\eta_{n}+\gamma_{n}}(y)-\Phi (y) \bigr\vert \leq C\bigl\{ \xi _{n}+\varepsilon_{1}+ \varepsilon_{2}+\mathrm{P}\bigl( \vert \eta_{n} \vert \geq \varepsilon_{1}\bigr)+\mathrm{P}\bigl( \vert \gamma_{n} \vert \geq \varepsilon_{2}\bigr)\bigr\} . $$

### Lemma 3.5

*Assume that* (A1)-(A5) *hold true*, *we can get that*

1. $\mathrm{E}(U_{1n}^{\prime \prime })^{2}\leq C\xi_{1n}$, $\mathrm{E}(U _{1n}^{\prime \prime \prime })^{2}\leq C\xi_{2n}$, $\mathrm{E}(U_{2n})^{2} \leq C\xi_{3n}$;
2. $\mathrm{P}( \vert U''_{1n} \vert \geq \xi_{1n}^{1/3}) \leq C \xi_{1n}^{1/3}$, $\mathrm{P}( \vert U'''_{1n} \vert \geq \xi_{2n}^{1/3}) \leq C \xi_{2n}^{1/3}$, $\mathrm{P}( \vert U_{2n} \vert \geq \xi_{3n}^{1/3}) \leq C \xi_{3n}^{1/3}$.

### Proof of Lemma 3.5

By Lemma 3.2 and assumptions (A4)-(A5), we can obtain that

$$\begin{aligned}& \begin{aligned} {\mathrm{E}}\bigl(U_{1n}^{\prime \prime }\bigr)^{2} &= \mathrm{E} \Biggl( \sum_{v=1}^{k} \sum _{i=l_{v}}^{l_{v}+q-1} \rho_{n}^{-1} \sum_{j=\max \{1,i-n\}}^{\min \{n,i+n\}}a_{j-i} \frac{t_{j}-t_{j-1}}{h _{n}}K \biggl( \frac{t-t_{j}}{h_{n}} \biggr) e_{i} \Biggr) ^{2} \\ &\leq C kq \biggl( \rho_{n}^{-1}\frac{t_{j}-t_{j-1}}{h_{n}}K \biggl( \frac{t-t _{j}}{h_{n}} \biggr) \biggr) ^{2} \Biggl( \sum _{j=\max \{1,i-n\}} ^{\min \{n,i+n\}} \vert a_{j-i} \vert \Biggr) ^{2} \\ &\leq C kqn^{-\theta } \Biggl( \sum_{j=-\infty }^{\infty } \vert a _{j} \vert \Biggr) ^{2}\leq C n^{1-\theta }qp^{-1} = C\xi_{1n}, \end{aligned} \\ & \begin{aligned} {\mathrm{E}}\bigl(U_{1n}^{\prime \prime \prime }\bigr)^{2} &= \mathrm{E} \Biggl( \sum_{i=k(p+q)-n+1}^{2n} \rho_{n}^{-1} \sum_{j=\max \{1,i-n \}}^{\min \{n,i+n\}}a_{j-i} \frac{t_{j}-t_{j-1}}{h_{n}}K \biggl( \frac{t-t _{j}}{h_{n}} \biggr) e_{i} \Biggr) ^{2} \\ &\leq C \bigl[3n-k(p+q)\bigr] \biggl( \rho_{n}^{-1} \frac{t_{j}-t_{j-1}}{h_{n}}K \biggl( \frac{t-t_{j}}{h_{n}} \biggr) \biggr) ^{2} \Biggl( \sum_{j=\max \{1,i-n\}}^{\min \{n,i+n\}} \vert a_{j-i} \vert \Biggr) ^{2} \\ &\leq C \bigl[3n-k(p+q)\bigr]n^{-\theta } \Biggl( \sum _{j=-\infty }^{ \infty } \vert a_{j} \vert \Biggr) ^{2} \leq C p n^{-\theta } = C\xi_{2n}, \end{aligned} \\ & {\mathrm{E}}\bigl(U_{2n}^{2}\bigr) = \mathrm{E} \Biggl\vert \rho_{n}^{-1}\sum_{i_{1}=1} ^{n}\frac{t_{i_{1}}-t_{i_{1}-1}}{h_{n}}K \biggl( \frac{t-t_{i_{1}}}{h _{n}} \biggr) \sum _{ \vert j_{1} \vert >n}a_{j_{1}}e_{i_{1}-j_{1}} \Biggr\vert \\ & \hphantom{{\mathrm{E}}\bigl(U_{2n}^{2}\bigr) } \quad{} \times \Biggl\vert \rho_{n}^{-1}\sum _{i_{2}=1}^{n}\frac{t_{i_{2}}-t _{i_{2}-1}}{h_{n}}K \biggl( \frac{t-t_{i_{2}}}{h_{n}} \biggr) \sum_{ \vert j_{2} \vert >n}a_{j_{2}}e_{i_{2}-j_{2}} \Biggr\vert \\ & \hphantom{{\mathrm{E}}\bigl(U_{2n}^{2}\bigr) }\leq C\mathrm{E} \Biggl\{ \sum_{i_{1}=1}^{n} \frac{t_{i_{1}}-t _{i_{1}-1}}{h_{n}}K \biggl( \frac{t-t_{i_{1}}}{h_{n}} \biggr) \sum _{i_{2}=1}^{n} \biggl\vert \sum _{ \vert j_{1} \vert >n}a_{j_{1}}e_{i_{1}-j _{1}} \biggr\vert \biggl\vert \sum_{ \vert j_{2} \vert >n}a_{j_{2}}e_{i_{2}-j_{2}} \biggr\vert \Biggr\} \\ & \hphantom{{\mathrm{E}}\bigl(U_{2n}^{2}\bigr) }\leq C\mathrm{E} \Biggl\{ \int^{+\infty }_{-\infty } \bigl\vert K(u) \bigr\vert \,du\sum _{i_{2}=1}^{n} \biggl\vert \sum _{ \vert j_{1} \vert >n}a_{j_{1}}e_{i_{1}-j _{1}} \biggr\vert \biggl\vert \sum_{ \vert j_{2} \vert >n}a_{j_{2}}e_{i_{2}-j_{2}} \biggr\vert \Biggr\} \\ & \hphantom{{\mathrm{E}}\bigl(U_{2n}^{2}\bigr) } \leq Cn \biggl( \sum_{ \vert j \vert >n} \vert a_{j} \vert \biggr) ^{2} = C\xi_{3n}. \end{aligned}$$

This completes the proof of Lemma 3.5(1). In addition, Lemma 3.5(2) can be derived from the Markov inequality and Lemma 3.5(1) immediately. □

### Lemma 3.6

*Assume that* (A1)-(A5) *hold*, *write* $u_{n}^{2}= \sum_{v=1}^{k}\operatorname {Var}(z_{nv})$, *we can get that*

$$ \bigl\vert u_{n}^{2}-1 \bigr\vert \leq C \bigl( \xi_{1n}^{1/2}+\xi_{2n}^{1/2}+ \xi_{3n}^{1/2}+ V(q) \bigr). $$

Let $\{\mu_{nv}:v=1,\ldots, k\}$ be independent random variables and $\mu_{nv}\stackrel{\mathcal{D}}{=}z_{nv}$, $v=1,\ldots, k$. Write $H_{n}=\sum_{v=1}^{k}{\mu_{nv}}$. Then we get the following.

### Proof of Lemma 3.6

Let $\Theta_{n}=\sum_{1\leq i< j\leq k} \operatorname {Cov}(z_{ni}, z_{nj})$, then $u_{n}^{2}=\mathrm{E}(U_{1n}^{\prime })^{2}-2\Theta_{n}$. By ${\mathrm{E}(U_{n})^{2}=1}$, Lemma 3.5(1), the $C_{r}$-inequality and the Cauchy-Schwarz inequality, it follows that

$$\begin{aligned}& \begin{aligned} {\mathrm{E}}\bigl(U^{\prime }_{1n}\bigr)^{2}&=\mathrm{E} \bigl[U_{n}-\bigl(U^{\prime \prime }_{1n}+U ^{\prime \prime \prime }_{1n}+U_{2n} \bigr)\bigr]^{2} \\ &=1+\mathrm{E}\bigl(U^{\prime \prime }_{1n}+U^{\prime \prime \prime }_{1n}+U_{2n} \bigr)^{2}-2{\mathrm{E}}\bigl(U_{n}\bigl(U ^{\prime \prime }_{1n}+U^{\prime \prime \prime }_{1n}+U_{2n} \bigr)\bigr), \end{aligned} \\& {\mathrm{E}}\bigl(U^{\prime \prime }_{1n}+U^{\prime \prime \prime }_{1n}+U_{2n} \bigr)^{2} \leq 2\bigl(\mathrm{E}\bigl(U^{\prime \prime }_{1n} \bigr)^{2}+\mathrm{E}\bigl(U^{\prime \prime \prime }_{1n} \bigr)^{2}+\mathrm{E}(U_{2n})^{2}\bigr)\leq C( \xi_{1n}+\xi_{2n}+\xi _{3n}), \\& {\mathrm{E}}\bigl(U_{n}\bigl(U^{\prime \prime }_{1n}+U^{\prime \prime \prime }_{1n}+U _{2n}\bigr)\bigr)\leq \sqrt{\mathrm{E}U^{2}_{n}} \sqrt{\mathrm{E}\bigl(U^{\prime \prime }_{1n}+U^{\prime \prime \prime }_{1n}+U_{2n} \bigr)^{2}}\leq C \bigl( \xi^{{1}/ {2}}_{1n}+ \xi^{{1}/{2}}_{2n}+\xi^{{1}/{2}}_{3n} \bigr) . \end{aligned}$$

Hence, it has been found that

3.1 $$ \begin{aligned}[b] \bigl\vert {\mathrm{E}}\bigl(U_{1n}^{\prime } \bigr)^{2}-1 \bigr\vert &= \bigl\vert {\mathrm{E}} \bigl(U_{1n}^{\prime \prime } +U_{1n}^{\prime \prime \prime }+U_{2n} \bigr)^{2} - 2{\mathrm{E}}\bigl(U_{n}\bigl(U_{1n} ^{\prime \prime }+U_{1n}^{\prime \prime \prime }+U_{2n}\bigr)\bigr) \bigr\vert \\ &\leq C \bigl( \xi_{1n}^{1/2}+\xi_{2n}^{1/2}+ \xi_{3n}^{1/2} \bigr) . \end{aligned} $$

On the other hand, from the basic definition of LNQD sequence, Lemma 3.1, (A1) and (A4), we can prove that

3.2 $$\begin{aligned} \vert \Theta_{n} \vert \leq & \sum_{1\leq i< j\leq k} \sum_{s_{1}=k_{i}}^{k_{i}+p-1} \sum _{t_{1}=k_{j}}^{k_{j}+p-1} { \bigl\vert \operatorname {Cov}(X_{ns_{1}}, X_{nt_{1}}) \bigr\vert } \\ \leq & \sum_{1\leq i< j\leq k}\sum _{s_{1}=k_{i}}^{k_{i}+p-1} \sum_{t_{1}=k_{j}}^{k_{j}+p-1} \sum_{w=\max \{1,s_{1}-n \}}^{\min \{n,s_{1}+n\}} \sum _{u=\max \{1,t_{1}-n\}}^{\min \{n,t _{1}+n\}}\rho_{n}^{-2} \biggl\vert \frac{t_{w}-t_{w-1}}{h_{n}}K \biggl( \frac{t-t _{w}}{h_{n}} \biggr) \\ & {}\times\frac{t_{u}-t_{u-1}}{h_{n}}K \biggl( \frac{t-t_{u}}{h_{n}} \biggr) \biggr\vert \vert a _{u-s_{1}}a_{v-t_{1}} \vert \bigl\vert \operatorname {Cov}(e_{s_{1}},e_{t_{1}}) \bigr\vert \\ \leq & C \sum_{i=1}^{k-1}\sum _{s_{1}=k_{i}}^{k_{i}+p-1} \sum_{w=\max \{1,s_{1}-n\}}^{\min \{n,s_{1}+n\}} \biggl\vert \frac{t _{w}-t_{w-1}}{h_{n}}K \biggl( \frac{t-t_{w}}{h_{n}} \biggr) \biggr\vert \vert a_{w-s _{1}} \vert \\ & {}\times \sum_{j=i+1}^{k} \sum_{t_{1}=k_{j}}^{k_{j}+p-1} \sum _{v=\max \{1,t_{1}-n\}}^{\min \{n,t_{1}+n\}} \vert a_{u-t_{1}} \vert \operatorname {Cov}(e_{s_{1}},e_{t_{1}}) \\ \leq & C \sum_{i=1}^{k-1}\sum _{s_{1}=k_{i}}^{k_{i}+p-1} \sum_{w=\max \{1,s_{1}-n\}}^{\min \{n,s_{1}+n\}} \biggl\vert \frac{t _{w}-t_{w-1}}{h_{n}}K \biggl( \frac{t-t_{w}}{h_{n}} \biggr) \biggr\vert \vert a_{w-s _{1}} \vert \\ & {}\times \sup_{s_{1}\geq 1}\sum _{t_{1}: \vert t_{1}-s_{1} \vert \geq q} \operatorname {Cov}(e_{s_{1}},e_{t_{1}})| \\ \leq & C V(q)\sum_{i=1}^{k-1}\sum _{s_{1}=k_{i}}^{k_{i}+p-1} \sum_{w=1}^{n} \biggl\vert \frac{t_{w}-t_{w-1}}{h_{n}}K \biggl( \frac{t-t _{w}}{h_{n}} \biggr) \biggr\vert \vert a_{w-s_{1}} \vert \\ \leq & C V(q)\sum_{w=1}^{n} \biggl\vert \frac{t_{w}-t_{w-1}}{h_{n}}K \biggl( \frac{t-t_{w}}{h_{n}} \biggr) \biggr\vert \Biggl( \sum _{i=1}^{k-1} \sum _{s_{1}=k_{i}}^{k_{i}+p-1} \vert a_{w-s_{1}} \vert \Biggr) \\ \leq & C V(q) \int^{+\infty }_{-\infty } \bigl\vert K(u) \bigr\vert \,du \Biggl( \sum_{i=1} ^{k-1}\sum _{s_{1}=k_{i}}^{k_{i}+p-1} \vert a_{u-s_{1}} \vert \Biggr) \leq C V(q). \end{aligned}$$

Therefore, combining equations (3.1) and (3.2), we can get that

$$ \bigl\vert u_{n}^{2}-1 \bigr\vert \leq \bigl\vert { \mathrm{E}} \bigl(U_{1n}^{\prime }\bigr)^{2}-1 \bigr\vert +2 \vert \Theta_{n} \vert \leq C \bigl( \xi_{1n}^{1/2} +\xi_{2n}^{1/2}+ \xi_{3n}^{1/2}+ V(q) \bigr) . $$

 □

### Lemma 3.7

*Assume that* (A1)-(A5) *hold true and*, *applying these in Lemma *3.6, *we can obtain that*

$$ \sup_{y} \bigl\vert {\mathrm{P}} ( H_{n}/u_{n} \leq y ) -\Phi (y) \bigr\vert \leq C\xi_{4n}. $$

### Proof of Lemma 3.7

By using the Berry-Esseen inequality [24], we obtain

3.3 $$ \sup_{y} \bigl\vert {\mathrm{P}} ( H_{n}/u_{n} \leq y ) -\Phi (y) \bigr\vert \leq C \frac{\sum_{v=1}^{k}{ \mathrm{E}} \vert z_{nv} \vert ^{r}}{u_{n}^{r}} \quad \text{for } r\geq 2. $$

According to Lemma 3.1, Lemma 3.2, (A1), (A4) and (A5), we can get that

3.4 $$\begin{aligned} \sum_{v=1}^{k} {\mathrm{E}} \vert z_{nv} \vert ^{r} =& \sum_{v=1}^{k} {\mathrm{E}} \Biggl\vert \sum_{j=k_{v}}^{k_{v}+p-1} \sum_{i=\max \{1,j-n\}}^{\min \{n,j+n\}} \rho_{n}^{-1}a_{i-j} \frac{t _{i}-t_{i-1}}{h_{n}}K \biggl( \frac{t-t_{i}}{h_{n}} \biggr) e_{j} \Biggr\vert ^{r} \\ \leq & Cp^{r/2-1}\rho_{n}^{-r}\sum _{v=1}^{k}\sum_{j=k _{v}}^{k_{v}+p-1} \sum_{i=\max \{1,j-n\}}^{\min \{n,j+n\}} \vert a _{i-j} \vert \biggl\vert \frac{t_{i}-t_{i-1}}{h_{n}}K \biggl( \frac{t-t_{i}}{h_{n}} \biggr) \biggr\vert \\ &{}\times \Biggl\vert \sum_{i=\max \{1,j-n\}}^{\min \{n,j+n\}}a_{i-j} \frac{t _{i}-t_{i-1}}{h_{n}}K \biggl( \frac{t-t_{i}}{h_{n}} \biggr) \Biggr\vert ^{r-1} \\ \leq & Cp^{r/2-1}\rho_{n}^{-r}\sum _{i=1}^{n} \biggl\vert \frac{t_{i}-t _{i-1}}{h_{n}}K \biggl( \frac{t-t_{i}}{h_{n}} \biggr) \biggr\vert \Biggl( \sum_{v=1}^{k} \sum_{j=k_{v}}^{k_{v}+p-1} \vert a_{i-j} \vert \Biggr) \\ \leq & Cp^{r/2-1}\rho_{n}^{-r} \int^{+\infty }_{-\infty } \bigl\vert K(u) \bigr\vert \,du \Biggl( \sum_{v=1}^{k}\sum _{j=k_{v}}^{k_{v}+p-1} \vert a_{i-j} \vert \Biggr) \\ \leq & Cp^{r/2-1}n^{(1-r/2)\theta }=\xi_{4n}. \end{aligned}$$

Hence, by Lemma 3.6, combining equations (3.3) and (3.4), we can obtain Lemma 3.7. □

### Lemma 3.8

*Assume that* (A1)-(A5) *hold*, *and applying this in Lemma *3.4, *we can obtain that*

$$ \sup_{y} \bigl\vert {\mathrm{P}}\bigl(U_{1n}^{\prime } \leq y\bigr)-\mathrm{P}(H_{n}\leq y) \bigr\vert \leq C \bigl( \xi_{4n} + V^{1/3}(q)\bigr). $$

### Proof of Lemma 3.8

Suppose that $\chi (t)$ and $\lambda (t)$ are the characteristic functions of $U_{1n}^{\prime }$ and $H_{n}$. Therefore, it follows from Lemma 3.3, (A1) and (A4) that

$$\begin{aligned} \bigl\vert \chi (t)-\lambda (t) \bigr\vert =& \Biggl\vert {\mathrm{E}} \exp \Biggl( \mathbf{i}t \sum_{v=1}^{k} z_{nv} \Biggr) -\prod_{v=1}^{k} { \mathrm{E}} \exp ( \mathbf{i}t z_{nv} ) \Biggr\vert \\ \leq & 4t^{2} \sum_{1\leq i< j\leq k}\sum _{s_{1}=k_{i}} ^{k_{i}+p-1} \sum_{t_{1}=k_{j}}^{k_{j}+p-1}{ \bigl\vert \operatorname {Cov}(X_{ns _{1}}, X_{nt_{1}}) \bigr\vert } \\ \leq & 4t^{2} \sum_{1\leq i< j\leq k}\sum _{s_{1}=k_{i}} ^{k_{i}+p-1}\sum_{t_{1}=k_{j}}^{k_{j}+p-1} \sum_{u=\max \{1,s_{1}-n\}}^{\min \{n,s_{1}+n\}} \sum _{v=\max \{1,t_{1}-n\}}^{\min \{n,t_{1}+n\}}\rho_{n}^{-2} \\ &{}\times\biggl\vert \frac{t_{u}-t_{u-1}}{h_{n}}K \biggl( \frac{t-t_{u}}{h_{n}} \biggr) \cdot \frac{t_{v}-t_{v-1}}{h_{n}}K \biggl( \frac{t-t_{v}}{h_{n}} \biggr) \biggr\vert \cdot \vert a_{u-s_{1}}a_{v-t_{1}} \vert \\ & {}\times \bigl\vert \operatorname {Cov}(e_{s_{1}},e_{t_{1}}) \bigr\vert \\ \leq& Ct^{2}V(q). \end{aligned}$$

It is easily seen that

3.5 $$ \int_{-T}^{T} \biggl\vert \frac{\chi (t)-\lambda (t)}{t} \biggr\vert \,dt \leq C V(q)T ^{2}, $$

which implies

$$ {\mathrm{P}}(H_{n}\leq y)=\mathrm{P}({H_{n}}/{u_{n}} \leq {y}/{u_{n}}). $$

Consequently, from Lemma 3.7, it has been found

$$\begin{aligned}& \sup_{y} \bigl\vert {\mathrm{P}}(H_{n}\leq y+u)-\mathrm{P}(H_{n}\leq y) \bigr\vert \\& \quad = \sup_{y}{\mathrm{P}} \bigl\vert ({H_{n}}/{u_{n}} \leq {y+u}/{u_{n}})-\mathrm{P}( {H_{n}}/{u_{n}}\leq {y}/{u_{n}}) \bigr\vert \\& \quad \leq \sup_{y} \bigl\vert {\mathrm{P}}({H_{n}}/{u_{n}} \leq {y+u}/{u_{n}})- \Phi ({y+u}/{u_{n}}) \bigr\vert +\sup _{y} \bigl\vert \Phi ({y+u}/{u_{n}})-\Phi ( {y}/{u_{n}}) \bigr\vert \\& \quad\quad {}+\sup_{y} \bigl\vert {\mathrm{P}}({H_{n}}/{u_{n}} \leq {y}/{u_{n}})- \Phi ({y}/{u_{n}}) \bigr\vert \\& \quad \leq 2\sup_{y} \bigl\vert {\mathrm{P}}({H_{n}}/{u_{n}} \leq {y}/{u_{n}})- \Phi (y) \bigr\vert +\sup_{y} \bigl\vert \Phi ({y+u}/{u_{n}})-\Phi ({y}/{u_{n}}) \bigr\vert \\& \quad \leq C \bigl( p^{r/2-1}n^{(1-r/2)\theta }+{ \vert u \vert }/{u_{n}} \bigr) \leq C \bigl( p^{r/2-1}n^{(1-r/2)\theta }+ \vert u \vert \bigr) . \end{aligned}$$

Therefore

3.6 $$\begin{aligned}& T\sup_{y} \int_{ \vert u \vert \leq C/T} \bigl\vert {\mathrm{P}}(H_{n} \leq y+u)- \mathrm{P}(H_{n} \leq y ) \bigr\vert \,du \\& \quad \leq CT\sup _{y} \int_{ \vert u \vert \leq C/T}\bigl\{ p^{r/2-1}n^{(1-r/2)\theta }+ \vert u \vert \bigr\} \,du \\& \quad \leq C\bigl(p^{r/2-1}n^{(1-r/2)\theta }+1/T\bigr). \end{aligned}$$

Thus, combining equations (3.5) and (3.6), taking $T=V^{-1/3}(q) $, it suffices to prove that

$$\begin{aligned}& \sup_{y} \bigl\vert {\mathrm{P}}\bigl(U_{1n}^{\prime } \leq y\bigr)-\mathrm{P}(H_{n} \leq y) \bigr\vert \\& \quad \leq \int_{-T}^{T}{ \biggl\vert \frac{\chi (t)-\lambda (t)}{t} \biggr\vert \,dt} + T \sup_{y} \int_{ \vert u \vert \leq C/T} \bigl\vert {\mathrm{P}}(H_{n}\leq y+u) - \mathrm{P}(H_{n}\leq y) \bigr\vert \,du \\& \quad \leq C\bigl\{ V(q)T^{2} + p^{r/2-1}n^{(1-r/2)\theta }+1/T\bigr\} = C\bigl(\xi_{4n} + V^{1/3}(q)\bigr). \end{aligned}$$

 □

## Proofs of the main results

### Proof of Theorem 2.1

4.1 $$\begin{aligned}& \sup_{y} \bigl\vert {\mathrm{P}}\bigl(U^{\prime }_{1n} \leq y\bigr)-\Phi (y) \bigr\vert \\& \quad \leq \sup_{y} \bigl\vert {\mathrm{P}} \bigl(U^{\prime }_{1n}\leq y\bigr)-\mathrm{P}(H_{n} \leq y) \bigr\vert +\sup_{y} \bigl\vert {\mathrm{P}}(H_{n} \leq y)-\Phi ({y}/{u_{n}}) \bigr\vert + \sup_{y} \bigl\vert \Phi ({y}/{u_{n}})-\Phi (y) \bigr\vert \\& \quad := I_{1n}+I_{2n}+I_{3n}. \end{aligned}$$

According to Lemma 3.8, Lemma 3.7 and Lemma 3.6, it follows that

4.2 $$\begin{aligned}& I_{1n}\leq C\bigl\{ \xi_{4n} + V^{1/3}(q)\bigr\} , \end{aligned}$$

4.3 $$\begin{aligned}& I_{2n}=\sup_{y} \bigl\vert { \mathrm{P}}({H_{n}}/{u_{n}}\leq {y}/{u_{n}})- \Phi ({y}/{u_{n}}) \bigr\vert =\sup_{y} \bigl\vert { \mathrm{P}}({H_{n}}/{u_{n}}\leq y)- \Phi (y) \bigr\vert \leq C\xi_{4n}, \end{aligned}$$

4.4 $$\begin{aligned}& I_{3n}\leq C \bigl\vert u_{n}^{2}-1 \bigr\vert \leq C \bigl\{ \xi_{1n}^{1/2} +\xi_{2n}^{1/2}+ \xi_{3n}^{1/2}+ V(q) \bigr\} . \end{aligned}$$

Hence, by (4.1)-(4.4), we have that

4.5 $$ \sup_{y} \bigl\vert {\mathrm{P}}\bigl(U_{1n}^{\prime } \leq y\bigr)-\Phi (y) \bigr\vert \leq C \bigl\{ \xi _{1n}^{1/2}+ \xi_{2n}^{1/2}+\xi_{3n}^{1/2}+ \xi_{4n} + V^{1/3}(q) \bigr\} . $$

Thus, by Lemma 3.4, Lemma 3.5(2) and (4.5), it suffices to prove that

$$\begin{aligned} \sup_{y} \bigl\vert {\mathrm{P}}(U_{n}\leq y) - \Phi (y) \bigr\vert \leq & C \Biggl\{ \sup_{y} \bigl\vert {\mathrm{P}}\bigl(U_{1n}^{\prime }\leq y\bigr)-\Phi (y) \bigr\vert +\sum_{i=1}^{3} \xi_{in}^{1/3}+\mathrm{P}\bigl( \bigl\vert U_{1n}^{\prime \prime } \bigr\vert \geq \xi_{1n}^{1/3} \bigr) \\ &{}+ \mathrm{P}\bigl( \bigl\vert U_{1n}^{\prime \prime \prime } \bigr\vert \geq \xi_{2n}^{1/3}\bigr) +\mathrm{P}\bigl( \vert U_{2n} \vert \geq \xi_{3n}^{1/3}\bigr) \Biggr\} \\ \leq & C \bigl\{ \xi_{1n}^{1/3}+\xi_{2n}^{1/3}+ \xi_{3n}^{1/3}+ \xi _{4n} + V^{1/3}(q) \bigr\} . \end{aligned}$$

 □

### Proof of Corollary 2.1

By (A1) we can easily see that $V(q)\to 0$, therefore Corollary 2.1 holds. □

### Proof of Corollary 2.2

Let $p=[n^{\kappa }]$, $q=[n^{2 \kappa -1}]$. Taking $\kappa =\frac{2\theta \delta +\delta +3}{6+4 \delta }$, $0<\kappa <\theta $, we have by $r=3$ that

$$\begin{aligned}& \xi_{1n}^{1/3}=\xi_{2n}^{1/3}=O \bigl( n^{-\frac{\theta -\kappa }{3}} \bigr) =O \bigl( n^{-\frac{2\theta \delta +6\theta -\delta -3}{18+12\delta }} \bigr) , \\& \xi^{1/3}_{3n}=n^{-\frac{2\theta \delta +6\theta -\delta -3}{18+12 \delta }} \biggl( n^{\frac{2\theta \delta +6\theta +3\delta +3}{12+8 \delta }}\sum _{ \vert j \vert >n} \vert a_{j} \vert \biggr) ^{2/3} =O \bigl( n^{-\frac{2\theta \delta +6\theta -\delta -3}{18+12\delta }} \bigr) , \\& \xi_{4n}=O \bigl( n^{-(r/2-1)(\theta -\kappa )} \bigr) \leq O \bigl( n^{-\frac{ \theta -\kappa }{3}} \bigr) =O \bigl( n^{-\frac{2\theta \delta +6\theta -\delta -3}{18+12\delta }} \bigr) , \\& V^{1/3}(q)=O \bigl( \bigl( q^{-(r-2)(r+\delta )/2\delta } \bigr) ^{1/3} \bigr) =O \bigl( n^{-\frac{\theta -\kappa }{3}} \bigr) =O \bigl( n^{-\frac{2 \theta \delta +6\theta -\delta -3}{18+12\delta }} \bigr) . \end{aligned}$$

Therefore, the desired result is completed by Corollary 2.1 immediately. □
